# Pancreatic Cancer Susceptibility Loci and Their Role in Survival

**DOI:** 10.1371/journal.pone.0027921

**Published:** 2011-11-18

**Authors:** Cosmeri Rizzato, Daniele Campa, Nathalia Giese, Jens Werner, P. Sivaramakrishna Rachakonda, Rajiv Kumar, Michaela Schanné, William Greenhalf, Eithne Costello, Kay-tee Khaw, Tim J. Key, Afshan Siddiq, Justo Lorenzo-Bermejo, Barbara Burwinkel, John P. Neoptolemos, Markus W. Büchler, Jörg D. Hoheisel, Andrea Bauer, Federico Canzian

**Affiliations:** 1 German Cancer Research Center (DKFZ), Heidelberg, Germany; 2 Clinic for General, Visceral and Transplantation Surgery, University of Heidelberg, Heidelberg, Germany; 3 Pancreas Biomedical Research Unit and the Liverpool Experimental Cancer Medicine Centre, National Institute for Health Research, Liverpool, United Kingdom; 4 University of Cambridge School of Clinical Medicine, Cambridge, United Kingdom; 5 Cancer Epidemiology Unit, University of Oxford, Oxford, United Kingdom; 6 Imperial College, London, United Kingdom; 7 Institute of Medical Biometry and Informatics, University Hospital Heidelberg, Heidelberg, Germany; 8 Division Molecular Biology of Breast Cancer, Department of Gynecology and Obstetrics, University of Heidelberg, Heidelberg, Germany; Technische Universität München, Germany

## Abstract

Pancreatic cancer has one of the worst mortality rates of all cancers. Little is known about its etiology, particularly regarding inherited risk. The PanScan project, a genome-wide association study, identified several common polymorphisms affecting pancreatic cancer susceptibility. Single nucleotide polymorphisms (SNPs) in *ABO*, sonic hedgehog (*SHH*), telomerase reverse transcriptase (*TERT*), nuclear receptor subfamily 5, group A, member 2 (*NR5A2*) were found to be associated with pancreatic cancer risk. Moreover the scan identified loci on chromosomes 13q22.1 and 15q14, to which no known genes or other functional elements are mapped. We sought to replicate these observations in two additional, independent populations (from Germany and the UK), and also evaluate the possible impact of these SNPs on patient survival. We genotyped 15 SNPs in 690 cases of pancreatic ductal adenocarcinoma (PDAC) and in 1277 healthy controls. We replicated several associations between SNPs and PDAC risk. Furthermore we found that SNP rs8028529 was weakly associated with a better overall survival (OS) in both populations. We have also found that *NR5A2* rs12029406_T allele was associated with a shorter survival in the German population. In conclusion, we found that rs8028529 could be, if these results are replicated, a promising marker for both risk and prognosis for this lethal disease.

## Introduction

Pancreatic cancer is the fifth leading cause of cancer deaths in Europe and the eighth worldwide, with a five year relative survival of less than 5% [1]. No effective screening test for this malignancy exists, and metastatic disease is commonly present at initial diagnosis. Established risk factors include cigarette smoking, obesity or overweight, a medical history of diabetes type II, and family history of pancreatic cancer [2].

The PanScan project, a genome-wide association study (GWAS), recently identified various pancreatic cancer susceptibility loci. Several single nucleotide polymorphisms (SNPs) in the gene regions of *ABO*, sonic hedgehog (*SHH*), telomerase reverse transcriptase (*TERT*), nuclear receptor subfamily 5, group A, member 2 (*NR5A2*) were found to be associated with pancreatic cancer risk [3]. Statistically significant associations were found also with SNPs mapping to a region on chromosome 13q22.1 and a region on chromosome 15q14, where no known genes are mapped [3].

The genes to which several of the GWAS loci were mapped are biologically plausible candidates for involvement in pancreatic cancer. Several early studies reported an association between ABO blood type and gastrointestinal cancers, strongest for gastric cancer but also notable for pancreatic cancer [4], [5]. SHH plays a key role as a morphogenic factor and is related to the formation of various malignancies, including pancreatic cancer [6]. *NR5A2* encodes a nuclear receptor of the fushi tarazu (Ftz-F1) subfamily that interacts with β-catenin and is predominantly expressed in exocrine pancreas, liver, intestine and ovaries in adults. The *TERT* gene encodes the catalytic subunit of telomerase, essential for maintaining telomere ends. While telomerase activity cannot be detected in most normal tissues, it is seen in approximately 90% of human cancers [7]. The region of chromosome 5p15.33 where *TERT* maps has been identified in genome-wide association studies of a number of different cancers, including brain tumors, lung cancer, basal cell carcinoma, and melanoma. Although the region on 13q22.1 does not contain any known gene, it is frequently deleted in a spectrum of cancers, including pancreatic cancer [8], [9], [10].

The GWAS has convincingly shown association between several of these loci and pancreatic cancer risk. For others (SNPs in the *SHH* region and in a gene desert on chromosome 15q14), it also showed promising associations, although supported by less strong statistical evidence [3]. Therefore, we sought to replicate these observations in an additional, independent population of 690 cases of pancreatic ductal adenocarcinoma (PDAC), and we evaluated the possible impact of these SNPs on patient survival.

## Materials and Methods

### Ethics Statement

All participants signed an informed written consent. The study was approved by the ethical review boards of the institutions responsible for subject recruitment in each of the recruitment centres. The ethical committees were the following: South West Research Ethics Committee (Liverpool Subjects), Ethikkommission der Medizinischen Fakultät, Heidelberg (German subjects), Ethics commitee of the University of Oxford (Oxford subjects), Ethics commitee of the University of Cambridge (Cambridge subjects).

### Study population

Samples were collected from pancreatic cancer patients during surgery between December 1996 and September 2009, snap-frozen in liquid nitrogen directly after resection and subsequently stored at −80°C.

Detailed information on the control population is given elsewhere. Briefly, a total of 1141 healthy blood donors of German origin were recruited in 2004 at the Institute of Transfusion Medicine, Mannheim, Germany [11].

136 British controls were selected from people recruited in two cohorts in EPIC, an ongoing prospective cohort being carried out in ten European countries. The EPIC-Norfolk cohort (http://www.srl.cam.ac.uk/epic/) comprises over 30,000 individuals, ages 45 to 75 at recruitment, resident in Norfolk, East Anglia, and recruited from general practice registers between 1993 and 1997 [12]. The EPIC-Oxford cohort comprises 65,429 people aged 20 years and above and living in the UK recruited between 1993 and 1999 [13]. Characteristics of patients and controls are described in Table 1.

**Table 1:** Characteristics of patients and healthy controls included in this study.

	German		British	
	Cases	Controls	Cases	Controls
N	576	1141	114	136
**Gender**				
Female	243	324	45	55
Male	320	816	69	81
Missing	13	1	0	0
**Age at diagnosis/recruitment**				
Mean	63.9	55.01	63.59	60.42
(standard deviation)	9.9	11.12	9.78	9.31
Median	65	58	64	61
(25%–75%)	(58–71)	(50–63)	(57–72)	(53–68)
**Survival days**				
Mean	548.97		434.2	
(standard deviation)	517.5		549.3	
Median	387		305	
(25%–75%)	(172–750)		(161.5–490)	

### Selection of genes and polymorphisms

We selected 15 polymorphisms that were found to be associated with the risk of developing pancreatic cancer by a recent GWAS [3]. In each of the six regions identified by the GWAS, we selected the SNPs showing the strongest associations: we selected rs12029406, rs10919791, rs3790844 in the *NR5A2* gene; rs4635969 and rs401681 for the *TERT*/*CLPTM1L* region; rs172310, rs167020 for *SHH*; rs657152, rs505922, rs630014, rs495828 for *ABO*; rs9543325, rs9543325 at 13q22.1; rs8028529 at 15q14. More detailed information on the selected SNPs is given in Table 2.

**Table 2:** SNPs genotyped and their position in the genome.

SNP	Gene/Region	Chromosome	Position in the genome
rs12029406	*NR5A2*	1q32.1	198,172,451
rs10919791	*NR5A2*	1q32.1	198,231,791
rs3790844	*NR5A2*	1q32.1	198,274,055
rs3790843	*NR5A2*	1q32.1	198,277,447
rs4635969	*TERT, CLPTM1L*	5p15.33	1,361,552
rs401681	*TERT, CLPTM1L*	5p15.33	1,375,087
rs172310	*SHH*	7q36	155,308,388
rs167020	*SHH*	7q36	155,312,494
rs657152	*ABO*	9q34	135,129,086
rs505922	*ABO*	9q34	135,139,050
rs630014	*ABO*	9q34	135,139,543
rs495828	*ABO*	9q34	135,144,688
rs9543325	Gene desert	13q22.1	72,814,629
rs9564966	Gene desert	13q22.1	72,794,222
rs8028529	Gene desert	15q14	34,441,889

### DNA extraction and genotyping

DNA was extracted from frozen or paraffin-embedded pancreatic tissues of 690 patients with resected tumors (576 from Heidelberg, 114 from Liverpool) using the AllPrep Isolation Kit (Qiagen, Hilden, Germany) according to the manufacturer's protocol. Genotyping was performed using an allele-specific PCR-based KASPar SNP genotyping system (KBiosciences, Hoddesdon, UK) as recommended by the manufacturer. The order of DNAs from cases and controls was randomized on PCR plates in order to ensure that an equal number of cases could be analyzed simultaneously. Thermocycling was performed according to the manufacturer's instructions. Detection was performed using an ABI PRISM 7900 HT sequence detection system with SDS 2.2 software (Applied Biosystems, Foster City, CA, USA).

Genotyping for British controls was performed in the context of a genome-wide association study using the Human 660W-Quad BeadChip array according to manufacturer's instructions (Illumina, San Diego, CA, USA) at Imperial College.

### Statistical analysis

Hardy-Weinberg equilibrium was tested in the controls by the chi square test. Risk analysis was performed in a total of 690 PDAC cases and 1277 healthy controls. We used logistic regression for multivariate analyses to assess the main effects of the genetic polymorphism on pancreatic cancer risk using a co-dominant inheritance model. The most common allele in the controls was assigned as the reference category. All analyses were adjusted for age and gender.

For survival analysis, the median follow-up time was computed with censored observations only (20%), whereas the median survival time was calculated using data from all patients. Overall survival (OS) was defined as the time interval between diagnosis and death (uncensored observation) or the last date when the patient was still alive (censored observation, medium follow up time 1249 days). OS was evaluated using methods for censored survival time. In particular, risk of dying was estimated by hazard ratios (HR) and 95% confidence intervals (CI) in Cox proportional hazard models. All the analyses were performed with STATA software (StataCorp, College Station, TX, USA). For the survival analysis, in order to take into account the number of tests performed in this project, we calculated for each gene/region the number of effective independent variables, M_eff_, by use of the SNP Spectral Decomposition approach [14]. We obtained a gene-wide M_eff_ value for each gene and also a study-wide M_eff_ value, by adding up the gene M_eff_'s. For the replication analysis, since the assocations had already been convincingly shown in a GWAS, a correction for multiple testing is not necessary, therefore we used a threshold of 0.05 to confirm our findings.

## Results

In this study we sought to investigate two different endpoints: replication of the associations between 15 GWAS SNPs and the risk of developing pancreatic cancer, and an evaluation of the possible associations between the same SNPs and patient survival.

We genotyped 15 SNPs in 690 cases of PDAC and 1277 healthy controls. The average call rate was 97.20% (range 94.69%–98.79%). For 27 cases, both normal and tumor tissues were available and used for genotyping. No differences were observed (398 informative genotype comparisons). Approximately 10% of the samples were analyzed in duplicate, and the concordance rate of the genotypes was higher than 99%. The genotype distributions at all loci were in Hardy-Weinberg equilibrium in controls, with non-significant chi square values (using a threshold of p<0.05, data not shown).

The frequencies and distribution of the genotypes and the odds ratios for the association of each polymorphism with PDAC are described in Table S1. We were able to replicate several significant associations between the SNPs and PDAC risk. Table 3 shows the SNPs associated with pancreatic cancer risk in this study. The strongest association with an increased risk of PDAC we observed was with the C allele of the 9q34 rs9543325 SNP (OR_het_ 1.23, 95% CI 0.98–1.55, OR_hom_ 1.60, 95% CI 1.14–2.25, P_trend_ = 0.0023). For two SNPs in *NR5A2* (rs12029406, rs10919791), one in *SHH* (rs167020) and the 15q14 region SNP (rs rs8028529) no statistically significant association was detected. Figure S1 shows a summary of the replications in the study. Table S1 shows the distribution of each SNP genotyped in the study and the relative ORs in the two populations separately and together.

**Table 3:** Polymorphisms associated with pancreatic cancer risk.

Genotype	Cases^a^	Controls^a^	OR^b^	95% Ci^b^	p_value_	p_trend_
rs401681 | 5p15.33 | 1,375,087 | *TERT, CLPTM1L* c						
CC	185/661 (27.99%)	420/1267 (33.15%)	1	Ref.		**0.0139**
CT	336/661 (50.83%)	618/1267 (48.78%)	1.24	(0.98–1.58)	0.08	
TT	140/661 (21.18%)	229/1267 (18.07%)	1.38	(1.02–1.87)	**0.04**	
CT+TT	476/661 (72.01%)	847/1267 (66.85%)	1.28	(1.02–1.61)	**0.03**	
rs657152 | 9q34 | 135,129,086 | *ABO* c						
GG	199/686 (29.01%)	437/1255 (34.82%)	1	Ref.		**0.0449**
GT	357/686 (52.04%)	591/1255 (47.09%)	1.26	(0.99–1.59)	0.06	
TT	130/686 (18.95%)	227/1255 (18.09%)	1.27	(0.94–1.73)	0.12	
GT+TT	487/686 (70.99%)	818/1255 (65.18%)	1.26	(1.01–1.58)	0.04	
rs505922 | 9q34 | 135,139,050 | *ABO* c						
TT	205/667 (30.73%)	478/1264 (37.82%)	1	Ref.		**0.0081**
TC	348/667 (52.17%)	591/1264 (46.76%)	1.3	(1.03–1.64)	**0.03**	
CC	114/667 (17.09%)	195/1264 (15.43%)	1.36	(0.99–1.86)	0.06	
TC+CC	462/667 (69.27%)	786/1264 (62.18%)	1.31	(1.05–1.64)	**0.02**	
rs495828 | 9q34 | 135,144,688 | *ABO* c						
GG	342/664 (51.51%)	725/1255 (57.77%)	1	Ref.		**0.0215**
GT	276/664 (41.57%)	450/1255 (35.86%)	1.26	(1.02–1.57)	0.04	
TT	46/664 (6.93%)	80/1255 (6.37%)	1.31	(0.85–2.01)	0.23	
GT+TT	322/664 (48.49%)	530/1255 (42.23%)	1.27	(1.03–1.56)	0.03	
rs9543325 | 13q22.1 | 72,814,629 | Gene desert c						
TT	221/628 (35.19%)	518/1262 (41.05%)	1	Ref.		**0.0023**
TC	310/628 (49.36%)	601/1262 (47.62%)	1.23	(0.98–1.55)	0.08	
CC	97/628 (15.45%)	143/1262 (11.33%)	1.6	(1.14–2.25)	**0.01**	
TC+CC	407/628 (64.81%)	744/1262 (58.95%)	1.3	(1.04–1.62)	**0.02**	
rs9564966 | 13q22.1 | 72,794,222 | Gene desert c						
GG	292/684 (42.69%)	604/1268 (47.63%)	1	Ref.		**0.0079**
GA	307/684 (44.88%)	549/1268 (43.3%)	1.08	(0.87–1.34)	0.48	
AA	85/684 (12.43%)	115/1268 (9.07%)	1.63	(1.15–2.32)	**0.01**	
GA+AA	392/684 (57.31%)	664/1268 (52.37%)	1.17	(0.95–1.44)	0.14	

a Numbers may not add up to 100% of subjects due to genotyping failure. All samples that did not give a reliable result in the first round of genotyping were resubmitted to up to two additional rounds of genotyping. Data points that were still not filled after this procedure were left blank.

b OR: odds ratio; CI: confidence interval. Adjusted for age and gender. Significant associations (p<0.05) are reported in bold.

c SNP | Chromosome | Position on chromosome (referred to NCBI build 36) | Closest gene(s).

We investigated a possible association between the selected SNPs and patient survival. Median survival of cases was different in patients from Liverpool (305 days) and Heidelberg (387 days; Cox regression test, p = 10^−5^), therefore we conducted this analysis separately for the two populations. The results of this analysis are reported in Table 4 and Table S2.

**Table 4:** SNPs associated with patient survival.

SNP/ Center	Number of	Number of	Per-allele^b^		A/A vs. A/B^b^		A/A vs. B/B^b^		A/A vs. A/B+B/B^b^	
	subjects^a^	deaths^a^	HR (95% CI)	p	HR (95% CI)	p	HR (95% CI)	p	HR (95% CI)	p
rs12029406 **1q32.1 (** ***NR5A2*** **)**										
Heidelberg	503	418	1.13 (0.99–1.29)	0.07	1.22 (0.99–1.51)	0.06	1.23 (0.92–1.63)	0.16	1.23 (1.01–1.49)	**0.04**
Liverpool	99	85	0.92 (0.68–1.24)	0.58	1.00 (0.63–1.58)	1.00	0.79 (0.40–1.55)	0.49	0.94 (0.61–1.45)	0.78
rs8028529 **15q14 (gene desert)**										
Heidelberg	508	418	0.84 (0.71–0.99)	**0.04**	0.73 (0.59–0.91)	**0.01**	0.93 (0.62–1.41)	0.74	0.76 (0.62–0.93)	**0.01**
Liverpool	108	93	0.76 (0.55–1.05)	0.10	0.96 (0.61–1.51)	0.86	**0.40 (0.16–1.01)**	**0.05**	0.80 (0.52–1.22)	0.30

a Numbers may not add up to 100% of subjects due to genotyping failure. All samples that did not give a reliable result in the first round of genotyping were resubmitted to up to two additional rounds of genotyping. Data points that were still not filled after this procedure were left blank.

b HR: hazard ratio; CI: confidence interval. Age and gender did not show statistically significant association with survival, therefore were not used as adjustment variables. Associations approaching statistical significance are reported in bold.

We found that SNP rs8028529 (located on chromosome 15q14) was weakly associated with a better OS in both populations. In the German population, we found that the heterozygous carriers had a better survival (HR = 0.73, 95%CI 0.59–0.91, Pvalue = 0.01, median survival of heterozygotes = 440 days, median survival of homozygotes for the common allele = 346 days), while in the British population a better survival was observed in homozygous carriers of the variant allele (HR = 0.40, 95%CI 0.16–1.01, P = 0.05, median survival of homozygotes for the variant allele = 421 days, median survival of homozygotes for the common allele = 287 days). Analysing all samples together, adjusting by age, gender and recruitment center, we observed that the combined genotype (C/T + C/C) had a statistically significant association with better survival of PDAC: HR = 0.76 (95% CI 0.64–0.92) p = 0.004.

In the German population the carriers of at least one T allele of the rs12029406 SNP, which belongs to the *NR5A2* gene, showed a shorter survival time (HR = 1.23, 95%CI 1.01–1.49, P = 0.04, median survival of allele carriers = 359 days). Table 4 shows the results for these two SNPs while table S2 shows the results for all the SNPs.

We calculated M_eff_ values for each candidate gene/region separately and for the whole study (by adding the individual gene M_eff_ values;). The study-wide M_eff_ was 9.8. We therefore used a study-wide significance p-threshold of 0.05/9.8 = 0.0051. Using this threshold, no significant associations were observed between any of the polymorphisms genotyped and patients survival, with the exception of the T allele of rs8028529, in the pooled population, with better patients survival.

## Discussion

Pancreatic cancer is among the deadliest of cancers, with mortality rates approaching incidence rates [1], [15]. There is no effective curative treatment yet for pancreatic cancer. Surgery offers the only treatment option that significantly improves survival. Therefore, finding genetic variants associated with disease risk, progression and survival is of the utmost importance. Given that there are few known risk factors, improved diagnostics and a better understanding of the molecular pathogenesis of this disease are urgently needed.

We report the re-evaluation of 15 SNPs found to be associated with pancreatic cancer risk as reported in PanScan [3] and their possible involvement in patient survival. In this study, we were able to replicate six SNPs at a P_value_ of at least 0.05 (0.0449−0.0023). We could not replicate the other reported associations, although the allelic frequencies in our study subjects were comparable to those obtained in PanScan, and trends of risk went in the same direction as reported by PanScan. A possible explanation of our failure in replicating the associations may be due to insufficient statistical power.

The most important and novel finding of this manuscript is the fact that the C allele of SNP rs8028529, located in a gene desert on chromosome 15q14 is associated with better survival. This association reached statistical significance, at the conventional 0.05 threshold, in both populations we studied, although for the German cases significance was only observed for the heterozygous (C/T) carriers, while for the British cases it was observed for the homozygous carriers of the variant allele (C/C) only. An analysis of all samples combined, adjusting by age, gender and recruitment center, revealed that the combined genotype (C/T + C/C) had a statistically significant association with better survival of PDAC: HR = 0.76 (95% CI 0.64–0.92) p = 0.004. In PanScan, the association was found between the C allele and an increased risk of pancreatic cancer. It is very difficult to understand the biological mechanism that could explain these associations since the SNP is located in a gene desert. The nearest gene (located at a distance of about 500 kb) is myeloid ecotropic insertion site homeobox 2 (*MEIS2*), which is known to be expressed at high levels in the pancreas and in pancreatic cancer (data from the In Silico Transcriptomics database)[16]. This gene encodes a homeobox protein belonging to the TALE ('three amino acid loop extension') family of homeodomain-containing proteins. TALE homeobox proteins are highly conserved transcription regulators, and several members have been shown to be essential contributors to developmental programs. Recent studies have shown that MEIS proteins may also be involved in tumorigenesis, although the underlying mechanism is not yet clear [17], [18]. We speculate that the SNP could be situated in a regulatory region of the *MEIS2* gene that may modify its expression and in this way alter cancer risk and prognosis. A similar mechanism seems to be in place at the locus on chromosome 8q24, where SNPs associated with risk of several cancer types are located in a gene desert. Recent data suggest that one of those SNPs can affect the binding of a transcription factor that regulates the WNT pathway and possibly the *MYC* oncogene, which maps about 1 Mb downstream [19].

In this report we have also found that the *NR5A2* rs12029406_T allele is associated with a shorter survival, although only in in the German population. In a recent review Li and Abruzzese [20] point out why this receptor may play a role in pancreatic cancer. The authors report that it has been speculated that *NR5A2* contributes to diseases linked to pancreatic dysfunction, such as diabetes. For example NR5A2 plays an important role in transcriptional activation of the adiponectin gene [21], an adipocyte-secreted hormone, that has been proposed to be a biological link between obesity and increased risk of pancreatic cancer [22]. It is interesting to note that a *NR5A2* gene variant has been associated with excess BMI in a genome-wide association study [23]. SNPs in *NR5A2*, such as rs12029406, might modulate the receptor activity which in turn can modify the disease risk and survival.

Applying the correction for multiple testing the only association which is lower than the study-wise threshold of 0.0051 was shown by the combined analysis of the two populations for the T allele of rs8028529 and better survival. The results in the two populations are not identical, however all the HRs go in the same direction in both populations, i.e. a protective effect of the variant allele, although they reach statistical significance only in the heterozygotes in the population from Heidelberg and only in the homozygotes in the population from Liverpool. Indeed, by pooling together the two populations the effect of allele remains similar in terms of HR, but the statistical significance increases, as it is expected by increasing the numbers of the subjects in study. It is not immediate to explain this discrepancy: it can be that residual confounding factors mask the associations for the heterozygotes in the British and in the homozygotes in the Germans, or the difference in the survival between the two populations may contribute to masking the true association. Finally it can be that these associations are due to chance.

These results have to be taken with caution and further replications and functional studies are warranted. The fact that the polymorphism is located in a vastly unexplored gene desert region makes it difficult to really assess an immediate clinical impact for the finding reported in this study. These results if confirmed may prompt research on the molecular biology of the mechanism and this can ultimately further our understanding of the disease. A good example is the 8q24 hits, where following an original epidemiological observation, many studies have contributed to uncover the relationship between the SNPs of the region and the activation of the *MYC* gene [19]. From this point of view our study may be considered as a first preliminary step that could contribute to a better understanding of the disease and, in the long term, to the establishment of diagnostic and prognostic tools.

In conclusion, we present here the replication of six previously associated SNPs with pancreatic cancer risk and the first evidence of a possible involvement of rs8028529 in PDAC prognosis.

## Supporting Information

Figure S1 **Replication of associations between PanScan SNPs and risk of PDAC.**
(TIF)

Table S1 **Associations of SNPs on chromosome 1q32.1, 5p15.33, 7q36, 9q34, 13q22.1, 15q14 with risk of PDAC.**
(DOC)

Table S2 **Cox regression analysis for SNPs genotyped in PDAC cases and survival.**
(DOC)

## References

[1] Ferlay J, Parkin DM, Steliarova-Foucher E (2010). Estimates of cancer incidence and mortality in Europe in 2008.. Eur J Cancer.

[2] Anderson K, Mack T, Silverman D (2006). Cancer of the pancreas..

[3] Amundadottir L, Kraft P, Stolzenberg-Solomon RZ, Fuchs CS, Petersen GM (2009). Genome-wide association study identifies variants in the ABO locus associated with susceptibility to pancreatic cancer.. Nat Genet.

[4] Aird I, Bentall HH, Roberts JA (1953). A relationship between cancer of stomach and the ABO blood groups.. Br Med J.

[5] Aird I, Lee DR, Roberts JA (1960). ABO blood groups and cancer of oesophagus, cancerof pancreas, and pituitary adenoma.. Br Med J.

[6] Thayer SP, di Magliano MP, Heiser PW, Nielsen CM, Roberts DJ (2003). Hedgehog is an early and late mediator of pancreatic cancer tumorigenesis.. Nature.

[7] Kim NW, Piatyszek MA, Prowse KR, Harley CB, West MD (1994). Specific association of human telomerase activity with immortal cells and cancer.. Science.

[8] Baudis M, Cleary ML (2001). Progenetix.net: an online repository for molecular cytogenetic aberration data.. Bioinformatics.

[9] Chen C, Brabham WW, Stultz BG, Frierson HF, Barrett JC (2001). Defining a common region of deletion at 13q21 in human cancers.. Genes Chromosomes Cancer.

[10] Baudis M (2007). Genomic imbalances in 5918 malignant epithelial tumors: an explorative meta-analysis of chromosomal CGH data.. BMC Cancer.

[11] Gast A, Bermejo JL, Flohr T, Stanulla M, Burwinkel B (2007). Folate metabolic gene polymorphisms and childhood acute lymphoblastic leukemia: a case-control study.. Leukemia.

[12] Day N, Oakes S, Luben R, Khaw KT, Bingham S (1999). EPIC-Norfolk: study design and characteristics of the cohort. European Prospective Investigation of Cancer.. Br J Cancer.

[13] Davey GK, Spencer EA, Appleby PN, Allen NE, Knox KH (2003). EPIC-Oxford: lifestyle characteristics and nutrient intakes in a cohort of 33 883 meat-eaters and 31 546 non meat-eaters in the UK.. Public Health Nutr.

[14] Gao X, Starmer J, Martin ER (2008). A multiple testing correction method for genetic association studies using correlated single nucleotide polymorphisms.. Genet Epidemiol.

[15] Jemal A, Siegel R, Ward E, Hao Y, Xu J (2008). Cancer statistics, 2008.. CA Cancer J Clin.

[16] Kilpinen S, Autio R, Ojala K, Iljin K, Bucher E (2008). Systematic bioinformatic analysis of expression levels of 17,330 human genes across 9,783 samples from 175 types of healthy and pathological tissues.. Genome Biol.

[17] Doolan P, Clynes M, Kennedy S, Mehta JP, Germano S (2009). TMEM25, REPS2 and Meis 1: favourable prognostic and predictive biomarkers for breast cancer.. Tumour Biol.

[18] Milech N, Gottardo NG, Ford J, D'Souza D, Greene WK (2010). MEIS proteins as partners of the TLX1/HOX11 oncoprotein.. Leuk Res.

[19] Tuupanen S, Turunen M, Lehtonen R, Hallikas O, Vanharanta S (2009). The common colorectal cancer predisposition SNP rs6983267 at chromosome 8q24 confers potential to enhanced Wnt signaling.. Nat Genet.

[20] Li D, Abbruzzese JL (2010). New strategies in pancreatic cancer: emerging epidemiologic and therapeutic concepts.. Clin Cancer Res.

[21] Barb D, Williams CJ, Neuwirth AK, Mantzoros CS (2007). Adiponectin in relation to malignancies: a review of existing basic research and clinical evidence.. Am J Clin Nutr.

[22] Stolzenberg-Solomon RZ, Weinstein S, Pollak M, Tao Y, Taylor PR (2008). Prediagnostic adiponectin concentrations and pancreatic cancer risk in male smokers.. Am J Epidemiol.

[23] Fox CS, Heard-Costa N, Cupples LA, Dupuis J, Vasan RS (2007). Genome-wide association to body mass index and waist circumference: the Framingham Heart Study 100K project.. BMC Med Genet.

