# Supplementary figures and images for: Pancreatic Cancer Susceptibility Loci and Their Role in Survival
Source: PLoS One. 2011 Nov 18;6(11):e27921. doi: 10.1371/journal.pone.0027921 (PMC3220706; doi:10.1371/journal.pone.0027921)

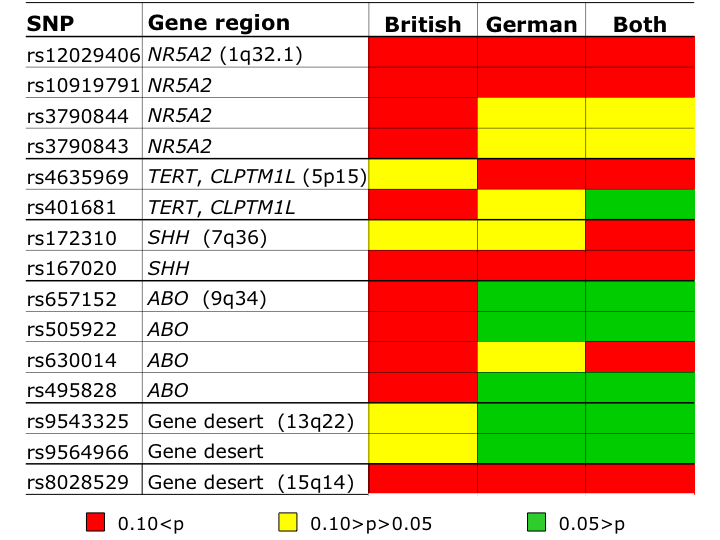

Supplement: Figure S1 — Replication of associations between PanScan SNPs and risk of PDAC. (TIF) [file pone.0027921.s001.tif]
