# Supplementary material for: Pancreatic Cancer Susceptibility Loci and Their Role in Survival
Source: PLoS One. 2011 Nov 18;6(11):e27921. doi: 10.1371/journal.pone.0027921 (PMC3220706; doi:10.1371/journal.pone.0027921)
Supplement: Table S1 — Associations of SNPs on chromosome 1q32.1, 5p15.33, 7q36, 9q34, 13q22.1, 15q14 with risk of PDAC. (DOC) [file pone.0027921.s002.doc]

**Supplementary table S1.** Replication of associations between PanScan SNPs and risk of PDAC.

| **Center** | **Genotype** | **Cases^a^** | **Controls^a^** | **OR^b^** | **95% Ci^b^** | **p_value_** | **p_trend_** |
| --- | --- | --- | --- | --- | --- | --- | --- |
| rs12029406 | 1q32.1 | 198,172,451 | *NR5A2* ^c^ | | | |  |  |  |  |
| Liverpool | CC | 45/99 (45.45%) | 55/136 (40.44%) | 1 | Ref. |  | 0.4971 |
|  | CT | 42/99 (42.42%) | 63/136 (46.32%) | 0.82 | (0.46-1.46) | 0.5 |  |
|  | TT | 12/99 (12.12%) | 18/136 (13.24%) | 0.78 | (0.33-1.81) | 0.56 |  |
|  | CT+TT | 54/99 (54.55%) | 81/136 (59.56%) | 0.81 | (0.47-1.39) | 0.45 |  |
| Heidelberg | CC | 217/541 (40.11%) | 406/1114 (36.45%) | 1 | Ref. |  | 0.1504 |
|  | CT | 242/541 (44.73%) | 521/1114 (46.77%) | 0.94 | (0.73-1.21) | 0.62 |  |
|  | TT | 82/541 (15.16%) | 187/1114 (16.79%) | 0.94 | (0.66-1.32) | 0.71 |  |
|  | CT+TT | 324/541 (59.89%) | 708/1114 (63.55%) | 0.94 | (0.74-1.19) | 0.59 |  |
| Both centers | CC | 262/640 (40.94%) | 461/1250 (36.88%) | 1 | Ref. |  | 0.0901 |
|  | CT | 284/640 (44.38%) | 584/1250 (46.72%) | 0.91 | (0.73-1.15) | 0.44 |  |
|  | TT | 94/640 (14.69%) | 205/1250 (16.4%) | 0.89 | (0.65-1.23) | 0.48 |  |
|  | CT+TT | 378/640 (59.06%) | 789/1250 (63.12%) | 0.91 | (0.73-1.13) | 0.38 |  |
| rs10919791 | 1q32.1 | 198,231,791 | *NR5A2* ^c^ | | | |  |  |  |  |
| Liverpool | GG | 70/110 (63.64%) | 87/136 (63.97%) | 1 | Ref. |  | 0.9009 |
|  | GA | 37/110 (33.64%) | 46/136 (33.82%) | 1.07 | (0.62-1.86) | 0.8 |  |
|  | AA | 3/110 (2.73%) | 3/136 (2.21%) | 1.29 | (0.25-6.77) | 0.76 |  |
|  | GA+AA | 40/110 (36.36%) | 49/136 (36.03%) | 1.09 | (0.64-1.86) | 0.76 |  |
| Heidelberg | GG | 335/510 (65.69%) | 705/1131 (62.33%) | 1 | Ref. |  | 0.2266 |
|  | GA | 155/510 (30.39%) | 378/1131 (33.42%) | 0.99 | (0.76-1.27) | 0.92 |  |
|  | AA | 20/510 (3.92%) | 48/1131 (4.24%) | 0.88 | (0.48-1.61) | 0.67 |  |
|  | GA+AA | 175/510 (34.31%) | 426/1131 (37.67%) | 0.97 | (0.76-1.24) | 0.83 |  |
| Both centers | GG | 405/620 (65.32%) | 792/1267 (62.51%) | 1 | Ref. |  | 0.2589 |
|  | GA | 192/620 (30.97%) | 424/1267 (33.46%) | 0.99 | (0.79-1.24) | 0.92 |  |
|  | AA | 23/620 (3.71%) | 51/1267 (4.03%) | 0.9 | (0.51-1.59) | 0.72 |  |
|  | GA+AA | 215/620 (34.68%) | 475/1267 (37.49%) | 0.98 | (0.78-1.22) | 0.85 |  |
| rs3790844 | 1q32.1 | 198,274,055 | *NR5A2* ^c^ | | | |  |  |  |  |
| Liverpool | TT | 71/109 (65.14%) | 85/136 (62.5%) | 1 | Ref. |  | 0.7139 |
|  | TC | 34/109 (31.19%) | 46/136 (33.82%) | 0.93 | (0.53-1.63) | 0.81 |  |
|  | CC | 4/109 (3.67%) | 5/136 (3.68%) | 1.09 | (0.28-4.34) | 0.9 |  |
|  | TC+CC | 38/109 (34.86%) | 51/136 (37.5%) | 0.95 | (0.55-1.62) | 0.84 |  |
| Heidelberg | TT | 343/534 (64.23%) | 671/1126 (59.59%) | 1 | Ref. |  | 0.1039 |
|  | TC | 166/534 (31.09%) | 398/1126 (35.35%) | 0.95 | (0.74-1.22) | 0.7 |  |
|  | CC | 25/534 (4.68%) | 57/1126 (5.06%) | 0.85 | (0.49-1.47) | 0.56 |  |
|  | TC+CC | 191/534 (35.77%) | 455/1126 (40.41%) | 0.94 | (0.74-1.19) | 0.6 |  |
| Both centers | TT | 414/643 (64.39%) | 756/1262 (59.9%) | 1 | Ref. |  | 0.0845 |
|  | TC | 200/643 (31.1%) | 444/1262 (35.18%) | 0.93 | (0.74-1.17) | 0.54 |  |
|  | CC | 29/643 (4.51%) | 62/1262 (4.91%) | 0.87 | (0.52-1.45) | 0.6 |  |
|  | TC+CC | 229/643 (35.61%) | 506/1262 (40.1%) | 0.92 | (0.74-1.15) | 0.47 |  |
| rs3790843 | 1q32.1 | 198,277,447 | *NR5A2* ^c^ | | | |  |  |  |  |
| Liverpool | GG | 60/110 (54.55%) | 72/136 (52.94%) | 1 | Ref. |  | 0.8143 |
|  | GA | 43/110 (39.09%) | 55/136 (40.44%) | 0.96 | (0.56-1.65) | 0.89 |  |
|  | AA | 7/110 (6.36%) | 9/136 (6.62%) | 0.93 | (0.32-2.69) | 0.89 |  |
|  | GA+AA | 50/110 (45.45%) | 64/136 (47.06%) | 0.96 | (0.57-1.61) | 0.87 |  |
| Heidelberg | GG | 324/574 (56.45%) | 594/1136 (52.29%) | 1 | Ref. |  | 0.0621 |
|  | GA | 216/574 (37.63%) | 454/1136 (39.96%) | 0.97 | (0.77-1.23) | 0.82 |  |
|  | AA | 34/574 (5.92%) | 88/1136 (7.75%) | 0.74 | (0.46-1.17) | 0.2 |  |
|  | GA+AA | 250/574 (43.55%) | 542/1136 (47.71%) | 0.93 | (0.74-1.17) | 0.55 |  |
| Both centers | GG | 384/684 (56.14%) | 666/1272 (52.36%) | 1 | Ref. |  | 0.0676 |
|  | GA | 259/684 (37.87%) | 509/1272 (40.02%) | 0.96 | (0.77-1.19) | 0.71 |  |
|  | AA | 41/684 (5.99%) | 97/1272 (7.63%) | 0.74 | (0.48-1.13) | 0.17 |  |
|  | GA+AA | 300/684 (43.86%) | 606/1272 (47.64%) | 0.92 | (0.75-1.14) | 0.45 |  |
| rs4635969 | 5p15.33 | 1,361,552 | *TERT, CLPTM1L* ^c^ | | | |  |  |  |  |
| Liverpool | CC | 81/110 (73.64%) | 86/136 (63.24%) | 1 | Ref. |  | 0.1031 |
|  | CT | 26/110 (23.64%) | 45/136 (33.09%) | 0.61 | (0.34-1.09) | 0.1 |  |
|  | TT | 3/110 (2.73%) | 5/136 (3.68%) | 0.69 | (0.16-3.08) | 0.63 |  |
|  | CT+TT | 29/110 (26.36%) | 50/136 (36.76%) | 0.62 | (0.35-1.08) | 0.09 |  |
| Heidelberg | CC | 334/525 (63.62%) | 709/1100 (64.45%) | 1 | Ref. |  | 0.6856 |
|  | CT | 165/525 (31.43%) | 341/1100 (31%) | 1.03 | (0.80-1.33) | 0.81 |  |
|  | TT | 26/525 (4.95%) | 50/1100 (4.55%) | 1.24 | (0.72-2.13) | 0.43 |  |
|  | CT+TT | 191/525 (36.38%) | 391/1100 (35.55%) | 1.06 | (0.83-1.35) | 0.65 |  |
| Both centers | CC | 415/635 (65.35%) | 795/1236 (64.32%) | 1 | Ref. |  | 0.7436 |
|  | CT | 191/635 (30.08%) | 386/1236 (31.23%) | 0.95 | (0.76-1.20) | 0.69 |  |
|  | TT | 29/635 (4.57%) | 55/1236 (4.45%) | 1.13 | (0.68-1.86) | 0.65 |  |
|  | CT+TT | 220/635 (34.65%) | 441/1236 (35.68%) | 0.97 | (0.78-1.22) | 0.82 |  |
| rs401681 | 5p15.33 | 1,375,087 | *TERT, CLPTM1L* ^c^ | | | |  |  |  |  |
| Liverpool | CC | 35/110 (31.82%) | 41/136 (30.15%) | 1 | Ref. |  | 0.4961 |
|  | CT | 46/110 (41.82%) | 70/136 (51.47%) | 0.73 | (0.40-1.34) | 0.31 |  |
|  | TT | 29/110 (26.36%) | 25/136 (18.38%) | 1.35 | (0.66-2.76) | 0.41 |  |
|  | CT+TT | 75/110 (68.18%) | 95/136 (69.85%) | 0.89 | (0.51-1.56) | 0.69 |  |
| Heidelberg | CC | 150/551 (27.22%) | 379/1131 (33.51%) | 1 | Ref. |  | **0.0204** |
|  | CT | 290/551 (52.63%) | 548/1131 (48.45%) | 1.36 | (1.04-1.77) | **0.02** |  |
|  | TT | 111/551 (20.15%) | 204/1131 (18.04%) | 1.39 | (1.00-1.94) | 0.05 |  |
|  | CT+TT | 401/551 (72.78%) | 752/1131 (66.49%) | 1.37 | (1.06-1.76) | **0.01** |  |
| Both centers | CC | 185/661 (27.99%) | 420/1267 (33.15%) | 1 | Ref. |  | **0.0139** |
|  | CT | 336/661 (50.83%) | 618/1267 (48.78%) | 1.24 | (0.98-1.58) | 0.08 |  |
|  | TT | 140/661 (21.18%) | 229/1267 (18.07%) | 1.38 | (1.02-1.87) | **0.04** |  |
|  | CT+TT | 476/661 (72.01%) | 847/1267 (66.85%) | 1.28 | (1.02-1.61) | **0.03** |  |
| rs172310 | 7q36 | 155,308,388 | *SHH* ^c^ | | |  |  |  |  |  |
| Liverpool | CC | 67/108 (62.04%) | 67/136 (49.26%) | 1 | Ref. |  | 0.0902 |
|  | CA | 30/108 (27.78%) | 52/136 (38.24%) | 0.59 | (0.33-1.05) | 0.07 |  |
|  | AA | 11/108 (10.19%) | 17/136 (12.5%) | 0.66 | (0.28-1.57) | 0.35 |  |
|  | CA+AA | 41/108 (37.96%) | 69/136 (50.74%) | 0.61 | (0.36-1.03) | 0.06 |  |
| Heidelberg | CC | 251/558 (44.98%) | 583/1124 (51.87%) | 1 | Ref. |  | 0.1023 |
|  | CA | 270/558 (48.39%) | 449/1124 (39.95%) | 1.26 | (1.00-1.60) | 0.06 |  |
|  | AA | 37/558 (6.63%) | 92/1124 (8.19%) | 0.85 | (0.54-1.35) | 0.5 |  |
|  | CA+AA | 307/558 (55.02%) | 541/1124 (48.13%) | 1.19 | (0.95-1.50) | 0.13 |  |
| Both centers | CC | 318/666 (47.75%) | 650/1260 (51.59%) | 1 | Ref. |  | 0.4331 |
|  | CA | 300/666 (45.05%) | 501/1260 (39.76%) | 1.14 | (0.91-1.41) | 0.25 |  |
|  | AA | 48/666 (7.21%) | 109/1260 (8.65%) | 0.83 | (0.55-1.24) | 0.36 |  |
|  | CA+AA | 348/666 (52.25%) | 610/1260 (48.41%) | 1.08 | (0.88-1.33) | 0.47 |  |
| rs167020 | 7q36 | 155,312,494 | *SHH* ^c^ | | |  |  |  |  |  |
| Liverpool | GG | 67/110 (60.91%) | 73/136 (53.68%) | 1 | Ref. |  | 0.1851 |
|  | GA | 34/110 (30.91%) | 46/136 (33.82%) | 0.85 | (0.48-1.50) | 0.57 |  |
|  | AA | 9/110 (8.18%) | 17/136 (12.5%) | 0.56 | (0.23-1.40) | 0.22 |  |
|  | GA+AA | 43/110 (39.09%) | 63/136 (46.32%) | 0.77 | (0.46-1.30) | 0.33 |  |
| Heidelberg | GG | 277/558 (49.64%) | 606/1141 (53.11%) | 1 | Ref. |  | 0.5642 |
|  | GA | 250/558 (44.8%) | 453/1141 (39.7%) | 1.1 | (0.87-1.39) | 0.43 |  |
|  | AA | 31/558 (5.56%) | 82/1141 (7.19%) | 0.76 | (0.47-1.24) | 0.28 |  |
|  | GA+AA | 281/558 (50.36%) | 535/1141 (46.89%) | 1.05 | (0.83-1.31) | 0.69 |  |
| Both centers | GG | 344/668 (51.5%) | 679/1277 (53.17%) | 1 | Ref. |  | 0.9759 |
|  | GA | 284/668 (42.51%) | 499/1277 (39.08%) | 1.06 | (0.86-1.32) | 0.57 |  |
|  | AA | 40/668 (5.99%) | 99/1277 (7.75%) | 0.73 | (0.48-1.12) | 0.15 |  |
|  | GA+AA | 324/668 (48.5%) | 598/1277 (46.83%) | 1.01 | (0.82-1.24) | 0.95 |  |
| rs657152 | 9q34 | 135,129,086 | *ABO* ^c^ | | |  |  |  |  |  |
| Liverpool | GG | 35/110 (31.82%) | 55/136 (40.44%) | 1 | Ref. |  | 0.4898 |
|  | GT | 60/110 (54.55%) | 59/136 (43.38%) | 1.43 | (0.81-2.54) | 0.21 |  |
|  | TT | 15/110 (13.64%) | 22/136 (16.18%) | 1.04 | (0.47-2.29) | 0.93 |  |
|  | GT+TT | 75/110 (68.18%) | 81/136 (59.56%) | 1.33 | (0.77-2.27) | 0.31 |  |
| Heidelberg | GG | 164/576 (28.47%) | 382/1119 (34.14%) | 1 | Ref. |  | **0.0424** |
|  | GT | 297/576 (51.56%) | 532/1119 (47.54%) | 1.23 | (0.95-1.59) | 0.12 |  |
|  | TT | 115/576 (19.97%) | 205/1119 (18.32%) | 1.33 | (0.96-1.85) | 0.09 |  |
|  | GT+TT | 412/576 (71.53%) | 737/1119 (65.86%) | 1.26 | (0.98-1.61) | 0.07 |  |
| Both centers | GG | 199/686 (29.01%) | 437/1255 (34.82%) | 1 | Ref. |  | **0.0449** |
|  | GT | 357/686 (52.04%) | 591/1255 (47.09%) | 1.26 | (0.99-1.59) | 0.06 |  |
|  | TT | 130/686 (18.95%) | 227/1255 (18.09%) | 1.27 | (0.94-1.73) | 0.12 |  |
|  | GT+TT | 487/686 (70.99%) | 818/1255 (65.18%) | 1.26 | (1.01-1.58) | 0.04 |  |
| rs505922 | 9q34 | 135,139,050 | *ABO* ^c^ | | |  |  |  |  |  |
| Liverpool | TT | 38/109 (34.86%) | 60/136 (44.12%) | 1 | Ref. |  | 0.3126 |
|  | TC | 58/109 (53.21%) | 59/136 (43.38%) | 1.4 | (0.81-2.45) | 0.23 |  |
|  | CC | 13/109 (11.93%) | 17/136 (12.5%) | 1.18 | (0.51-2.74) | 0.7 |  |
|  | TC+CC | 71/109 (65.14%) | 76/136 (55.88%) | 1.35 | (0.80-2.30) | 0.26 |  |
| Heidelberg | TT | 167/558 (29.93%) | 418/1128 (37.06%) | 1 | Ref. |  | **0.0084** |
|  | TC | 290/558 (51.97%) | 532/1128 (47.16%) | 1.29 | (1.00-1.66) | 0.05 |  |
|  | CC | 101/558 (18.1%) | 178/1128 (15.78%) | 1.4 | (0.99-1.97) | 0.06 |  |
|  | TC+CC | 391/558 (70.07%) | 710/1128 (62.94%) | 1.31 | (1.03-1.67) | **0.03** |  |
| Both centers | TT | 205/667 (30.73%) | 478/1264 (37.82%) | 1 | Ref. |  | **0.0081** |
|  | TC | 348/667 (52.17%) | 591/1264 (46.76%) | 1.3 | (1.03-1.64) | **0.03** |  |
|  | CC | 114/667 (17.09%) | 195/1264 (15.43%) | 1.36 | (0.99-1.86) | 0.06 |  |
|  | TC+CC | 462/667 (69.27%) | 786/1264 (62.18%) | 1.31 | (1.05-1.64) | **0.02** |  |
| rs630014 | 9q34 | 135,139,543 | *ABO* ^c^ | | |  |  |  |  |  |
| Liverpool | CC | 33/114 (28.95%) | 37/136 (27.21%) | 1 | Ref. |  | 0.3281 |
|  | CT | 62/114 (54.39%) | 67/136 (49.26%) | 1.15 | (0.63-2.10) | 0.65 |  |
|  | TT | 19/114 (16.67%) | 32/136 (23.53%) | 0.76 | (0.36-1.62) | 0.48 |  |
|  | CT+TT | 81/114 (71.05%) | 99/136 (72.79%) | 1.03 | (0.58-1.82) | 0.93 |  |
| Heidelberg | CC | 193/567 (34.04%) | 346/1123 (30.81%) | 1 | Ref. |  | 0.2361 |
|  | CT | 279/567 (49.21%) | 578/1123 (51.47%) | 0.8 | (0.62-1.03) | 0.08 |  |
|  | TT | 95/567 (16.75%) | 199/1123 (17.72%) | 0.84 | (0.60-1.18) | 0.31 |  |
|  | CT+TT | 374/567 (65.96%) | 777/1123 (69.19%) | 0.81 | (0.63-1.03) | 0.08 |  |
| Both centers | CC | 226/681 (33.19%) | 383/1259 (30.42%) | 1 | Ref. |  | 0.1815 |
|  | CT | 341/681 (50.07%) | 645/1259 (51.23%) | 0.86 | (0.68-1.09) | 0.21 |  |
|  | TT | 114/681 (16.74%) | 231/1259 (18.35%) | 0.83 | (0.61-1.13) | 0.24 |  |
|  | CT+TT | 455/681 (66.81%) | 876/1259 (69.58%) | 0.85 | (0.68-1.07) | 0.16 |  |
| rs495828 | 9q34 | 135,144,688 | *ABO* ^c^ | | |  |  |  |  |  |
| Liverpool | GG | 65/110 (59.09%) | 73/136 (53.68%) | 1 | Ref. |  | 0.2139 |
|  | GT | 39/110 (35.45%) | 49/136 (36.03%) | 0.84 | (0.48-1.46) | 0.53 |  |
|  | TT | 6/110 (5.45%) | 14/136 (10.29%) | 0.5 | (0.18-1.40) | 0.19 |  |
|  | GT+TT | 45/110 (40.91%) | 63/136 (46.32%) | 0.76 | (0.45-1.29) | 0.31 |  |
| Heidelberg | GG | 277/554 (50%) | 652/1119 (58.27%) | 1 | Ref. |  | **0.0026** |
|  | GT | 237/554 (42.78%) | 401/1119 (35.84%) | 1.35 | (1.07-1.72) | **0.01** |  |
|  | TT | 40/554 (7.22%) | 66/1119 (5.9%) | 1.64 | (1.02-2.64) | **0.04** |  |
|  | GT+TT | 277/554 (50%) | 467/1119 (41.73%) | 1.39 | (1.11-1.75) | **0.01** |  |
| Both centers | GG | 342/664 (51.51%) | 725/1255 (57.77%) | 1 | Ref. |  | **0.0215** |
|  | GT | 276/664 (41.57%) | 450/1255 (35.86%) | 1.26 | (1.02-1.57) | **0.04** |  |
|  | TT | 46/664 (6.93%) | 80/1255 (6.37%) | 1.31 | (0.85-2.01) | 0.23 |  |
|  | GT+TT | 322/664 (48.49%) | 530/1255 (42.23%) | 1.27 | (1.03-1.56) | **0.03** |  |
| rs9543325 | 13q22.1 | 72,814,629 | Gene desert ^c^ | | | |  |  |  |  |
| Liverpool | TT | 33/108 (30.56%) | 57/136 (41.91%) | 1 | Ref. |  | 0.1384 |
|  | TC | 60/108 (55.56%) | 62/136 (45.59%) | 1.61 | (0.92-2.84) | 0.1 |  |
|  | CC | 15/108 (13.89%) | 17/136 (12.5%) | 1.39 | (0.60-3.24) | 0.44 |  |
|  | TC+CC | 75/108 (69.44%) | 79/136 (58.09%) | 1.57 | (0.91-2.69) | 0.1 |  |
| Heidelberg | TT | 188/520 (36.15%) | 461/1126 (40.94%) | 1 | Ref. |  | **0.0084** |
|  | TC | 250/520 (48.08%) | 539/1126 (47.87%) | 1.17 | (0.91-1.51) | 0.22 |  |
|  | CC | 82/520 (15.77%) | 126/1126 (11.19%) | 1.7 | (1.17-2.46) | **0.01** |  |
|  | TC+CC | 332/520 (63.85%) | 665/1126 (59.06%) | 1.27 | (1.00-1.61) | 0.05 |  |
| Both centers | TT | 221/628 (35.19%) | 518/1262 (41.05%) | 1 | Ref. |  | 0.0023 |
|  | TC | 310/628 (49.36%) | 601/1262 (47.62%) | 1.23 | (0.98-1.55) | 0.08 |  |
|  | CC | 97/628 (15.45%) | 143/1262 (11.33%) | 1.6 | (1.14-2.25) | 0.01 |  |
|  | TC+CC | 407/628 (64.81%) | 744/1262 (58.95%) | 1.3 | (1.04-1.62) | 0.02 |  |
| rs9564966 | 13q22.1 | 72,794,222 | Gene desert ^c^ | | | |  |  |  |  |
| Liverpool | GG | 40/109 (36.7%) | 65/136 (47.79%) | 1 | Ref. |  | 0.0905 |
|  | GA | 56/109 (51.38%) | 59/136 (43.38%) | 1.41 | (0.82-2.45) | 0.22 |  |
|  | AA | 13/109 (11.93%) | 12/136 (8.82%) | 1.71 | (0.70-4.21) | 0.24 |  |
|  | GA+AA | 69/109 (63.3%) | 71/136 (52.21%) | 1.46 | (0.87-2.47) | 0.16 |  |
| Heidelberg | GG | 252/575 (43.83%) | 539/1132 (47.61%) | 1 | Ref. |  | **0.0329** |
|  | GA | 251/575 (43.65%) | 490/1132 (43.29%) | 1.04 | (0.82-1.32) | 0.75 |  |
|  | AA | 72/575 (12.52%) | 103/1132 (9.1%) | 1.71 | (1.16-2.51) | **0.01** |  |
|  | GA+AA | 323/575 (56.17%) | 593/1132 (52.39%) | 1.14 | (0.91-1.43) | 0.25 |  |
| Both centers | GG | 292/684 (42.69%) | 604/1268 (47.63%) | 1 | Ref. |  | **0.0079** |
|  | GA | 307/684 (44.88%) | 549/1268 (43.3%) | 1.08 | (0.87-1.34) | 0.48 |  |
|  | AA | 85/684 (12.43%) | 115/1268 (9.07%) | 1.63 | (1.15-2.32) | **0.01** |  |
|  | GA+AA | 392/684 (57.31%) | 664/1268 (52.37%) | 1.17 | (0.95-1.44) | 0.14 |  |
| rs8028529 | 15q14 | 34,441,889 | Gene desert ^c^ | | | |  |  |  |  |
| Liverpool | TT | 68/108 (62.96%) | 81/136 (59.56%) | 1 | Ref. |  | 0.862 |
|  | TC | 33/108 (30.56%) | 49/136 (36.03%) | 0.8 | (0.46-1.41) | 0.45 |  |
|  | CC | 7/108 (6.48%) | 6/136 (4.41%) | 1.62 | (0.50-5.21) | 0.42 |  |
|  | TC+CC | 40/108 (37.04%) | 55/136 (40.44%) | 0.89 | (0.52-1.51) | 0.66 |  |
| Heidelberg | TT | 343/544 (63.05%) | 687/1141 (60.21%) | 1 | Ref. |  | 0.5716 |
|  | TC | 167/544 (30.7%) | 395/1141 (34.62%) | 0.92 | (0.72-1.18) | 0.52 |  |
|  | CC | 34/544 (6.25%) | 59/1141 (5.17%) | 1.39 | (0.84-2.29) | 0.2 |  |
|  | TC+CC | 201/544 (36.95%) | 454/1141 (39.79%) | 0.98 | (0.77-1.24) | 0.86 |  |
| Both centers | TT | 411/652 (63.04%) | 768/1277 (60.14%) | 1 | Ref. |  | 0.555 |
|  | TC | 200/652 (30.67%) | 444/1277 (34.77%) | 0.9 | (0.72-1.13) | 0.35 |  |
|  | CC | 41/652 (6.29%) | 65/1277 (5.09%) | 1.46 | (0.92-2.30) | 0.11 |  |
|  | TC+CC | 241/652 (36.96%) | 509/1277 (39.86%) | 0.96 | (0.78-1.19) | 0.73 |  |

^a^ Numbers may not add up to 100% of subjects due to genotyping failure. All samples that did not give a reliable result in the first round of genotyping were resubmitted to up to two additional rounds of genotyping. Data points that were still not filled after this procedure were left blank.

^b^ OR: odds ratio; CI: confidence interval. Adjusted for age and gender. Significant associations (p< 0.05) are reported in bold.

^c^ SNP | Chromosome | Position on chromosome (referred to NCBI build 36) | Closest gene(s)
