# Supplementary material for: Pancreatic Cancer Susceptibility Loci and Their Role in Survival
Source: PLoS One. 2011 Nov 18;6(11):e27921. doi: 10.1371/journal.pone.0027921 (PMC3220706; doi:10.1371/journal.pone.0027921)
Supplement: Table S2 — Cox regression analysis for SNPs genotyped in PDAC cases and survival. (DOC) [file pone.0027921.s003.doc]

**Supplementary table S2.** Cox regression analysis for SNPs genotyped in PDAC cases and survival.

| **SNP** | **Center** | **Number of**  **subjects^a^** | **Number of**  **deaths^a^** | **Per-allele^b^** |  | **A/A vs. A/B^b^** |  | **A/A vs. B/B^b^** |  | **A/A vs. A/B+B/B^b^** |  |
| --- | --- | --- | --- | --- | --- | --- | --- | --- | --- | --- | --- |
|  |  |  |  | **HR (95% CI)** | **p** | **HR (95% CI)** | **p** | **HR (95% CI)** | **p** | **HR (95% CI)** | **p** |
| **1q32.1 (*NR5A2*)** | | | | | | | | | | | |
| rs12029406 | Heidelberg | 158 | 121 | 1.02 (0.78-1.32) | 0.90 | 1.13 (0.77-1.67) | 0.54 | 0.96 (0.54-1.71) | 0.89 | 1.09 (0.75-1.58) | 0.65 |
|  | Liverpool | 99 | 85 | 0.92 (0.68-1.24) | 0.58 | 1.00 (0.63-1.58) | 1.00 | 0.79 (0.40-1.55) | 0.49 | 0.94 (0.61-1.45) | 0.78 |
| rs10919791 | Heidelberg | 172 | 130 | 0.98 (0.72-1.33) | 0.90 | 1.03 (0.72-1.48) | 0.85 | 0.79 (0.29-2.15) | 0.64 | 1.01 (0.71-1.44) | 0.96 |
|  | Liverpool | 110 | 95 | 0.80 (0.55-1.15) | 0.23 | 0.89 (0.58-1.37) | 0.60 | 0.39 (0.10-1.61) | 0.19 | 0.83 (0.54-1.26) | 0.39 |
| rs3790844 | Heidelberg | 173 | 130 | 0.91 (0.67-1.23) | 0.54 | 0.97 (0.68-1.38) | 0.86 | 0.65 (0.24-1.78) | 0.40 | 0.94 (0.66-1.33) | 0.71 |
|  | Liverpool | 109 | 94 | 0.82 (0.58-1.16) | 0.26 | 0.96 (0.62-1.48) | 0.84 | 0.43 (0.13-1.37) | 0.15 | 0.86 (0.56-1.32) | 0.49 |
| rs3790843 | Heidelberg | 174 | 131 | 0.89 (0.67-1.17) | 0.39 | 0.96 (0.68-1.37) | 0.83 | 0.65 (0.30-1.42) | 0.28 | 0.91 (0.65-1.29) | 0.61 |
|  | Liverpool | 110 | 95 | 0.90 (0.66-1.23) | 0.50 | 1.06 (0.70-1.62) | 0.78 | 0.61 (0.26-1.43) | 0.26 | 0.97 (0.64-1.45) | 0.87 |
| **5p15.33 (*TERT, CLPTM1L*)** | | | | | | | | | | | |
| rs4635969 | Heidelberg | 172 | 130 | 0.94 (0.70-1.26) | 0.67 | 0.90 (0.61-1.32) | 0.58 | 0.96 (0.44-2.08) | 0.92 | 0.91 (0.63-1.31) | 0.60 |
|  | Liverpool | 110 | 95 | 1.25 (0.83-1.89) | 0.28 | 1.09 (0.67-1.76) | 0.73 | 2.92 (0.91-9.41) | 0.07 | 1.18 (0.74-1.86) | 0.49 |
| rs401681 | Heidelberg | 173 | 131 | 0.90 (0.71-1.14) | 0.38 | 0.96 (0.64-1.42) | 0.83 | 0.80 (0.48-1.30) | 0.37 | 0.90 (0.62-1.31) | 0.60 |
|  | Liverpool | 110 | 95 | 1.14 (0.88-1.47) | 0.31 | 1.53 (0.95-2.49) | 0.08 | 1.28 (0.75-2.19) | 0.37 | 1.42 (0.92-2.22) | 0.12 |
| **7q36.3 (*SHH*)** | | | | | | | | | | | |
| rs172310 | Heidelberg | 173 | 131 | 0.85 (0.64-1.14) | 0.28 | 0.76 (0.53-1.09) | 0.14 | 0.89 (0.46-1.70) | 0.72 | 0.78 (0.55-1.10) | 0.16 |
|  | Liverpool | 108 | 93 | 0.84 (0.61-1.17) | 0.31 | 0.65 (0.40-1.06) | 0.09 | 0.96 (0.47-1.93) | 0.90 | 0.72 (0.47-1.11) | 0.13 |
| rs167020 | Heidelberg | 171 | 129 | 0.89 (0.66-1.19) | 0.43 | 0.77 (0.53-1.10) | 0.15 | 1.05 (0.54-2.07) | 0.88 | 0.80 (0.56-1.13) | 0.21 |
|  | Liverpool | 110 | 95 | 0.85 (0.60-1.20) | 0.35 | 0.68 (0.43-1.08) | 0.10 | 1.07 (0.49-2.36) | 0.86 | 0.74 (0.48-1.13) | 0.16 |
| **9q34 (*ABO*)** | | | | | | | | | | | |
| rs657152 | Heidelberg | 174 | 131 | 1.22 (0.92-1.61) | 0.17 | 1.13 (0.72-1.77) | 0.61 | 1.47 (0.85-2.54) | 0.16 | 1.20 (0.77-1.86) | 0.42 |
|  | Liverpool | 110 | 95 | 0.91 (0.67-1.22) | 0.53 | 0.88 (0.56-1.39) | 0.59 | 0.83 (0.45-1.55) | 0.57 | 0.87 (0.57-1.34) | 0.53 |
| rs505922 | Heidelberg | 174 | 131 | 1.31 (0.98-1.75) | 0.07 | 1.07 (0.70-1.64) | 0.76 | 1.77 (1.03-3.05) | 0.04 | 1.18 (0.78-1.79) | 0.44 |
|  | Liverpool | 109 | 94 | 0.92 (0.68-1.24) | 0.59 | 0.93 (0.60-1.45) | 0.75 | 0.84 (0.44-1.60) | 0.60 | 0.91 (0.60-1.38) | 0.65 |
| rs630014 | Heidelberg | 173 | 130 | 0.80 (0.61-1.05) | 0.10 | 0.79 (0.54-1.16) | 0.23 | 0.65 (0.37-1.13) | 0.13 | 0.76 (0.52-1.10) | 0.14 |
|  | Liverpool | 114 | 99 | 1.02 (0.74-1.40) | 0.89 | 0.78 (0.50-1.23) | 0.29 | 1.17 (0.65-2.12) | 0.60 | 0.86 (0.56-1.32) | 0.49 |
| rs495828 | Heidelberg | 170 | 127 | 1.04 (0.76-1.41) | 0.82 | 0.94 (0.65-1.35) | 0.73 | 1.38 (0.65-2.91) | 0.40 | 0.97 (0.68-1.39) | 0.88 |
|  | Liverpool | 110 | 95 | 0.79 (0.57-1.10) | 0.17 | 0.78 (0.51-1.21) | 0.28 | 0.64 (0.27-1.49) | 0.30 | 0.76 (0.50-1.15) | 0.19 |
| **13q22.1 (gene desert)** | | | | | | | | | | | |
| rs9564966 | Heidelberg | 170 | 129 | 0.84 (0.64-1.10) | 0.19 | 0.75 (0.51-1.11) | 0.15 | 0.74 (0.43-1.28) | 0.29 | 0.75 (0.52-1.09) | 0.13 |
|  | Liverpool | 108 | 93 | 0.81 (0.58-1.11) | 0.19 | 0.80 (0.51-1.25) | 0.32 | 0.65 (0.33-1.31) | 0.23 | 0.77 (0.49-1.19) | 0.23 |
| rs9543325 | Heidelberg | 173 | 130 | 0.90 (0.69-1.19) | 0.47 | 0.76 (0.52-1.11) | 0.15 | 0.95 (0.55-1.64) | 0.85 | 0.79 (0.56-1.13) | 0.21 |
|  | Liverpool | 109 | 94 | 0.77 (0.56-1.06) | 0.11 | 0.76 (0.49-1.17) | 0.21 | 0.60 (0.3-1.22) | 0.16 | 0.72 (0.48-1.10) | 0.13 |
| **15q14 (gene desert)** | | | | | | | | | | | |
| rs8028529 | Heidelberg | 174 | 131 | 0.82 (0.61-1.10) | 0.18 | **0.69 (0.47-1.01)** | **0.05** | 0.96 (0.48-1.91) | 0.90 | 0.73 (0.51-1.04) | 0.08 |
|  | Liverpool | 108 | 93 | 0.76 (0.55-1.05) | 0.10 | 0.96 (0.61-1.51) | 0.86 | **0.40 (0.16-1.01)** | **0.05** | 0.80 (0.52-1.22) | 0.30 |

^a^ Numbers may not add up to 100% of subjects due to genotyping failure. All samples that did not give a reliable result in the first round of genotyping were resubmitted to up to two additional rounds of genotyping. Data points that were still not filled after this procedure were left blank

^b^ HR: hazard ratio; CI: confidence interval. Age and gender did not show statistically significant association with survival, therefore were not used as adjustment variables. Associations approaching statistical significance are reported in bold
